# Weather Forecasting by Insects: Modified Sexual Behaviour in Response to Atmospheric Pressure Changes

**DOI:** 10.1371/journal.pone.0075004

**Published:** 2013-10-02

**Authors:** Ana Cristina Pellegrino, Maria Fernanda Gomes Villalba Peñaflor, Cristiane Nardi, Wayne Bezner-Kerr, Christopher G. Guglielmo, José Maurício Simões Bento, Jeremy N. McNeil

**Affiliations:** 1 Department of Entomology and Acarology, University of São Paulo, Escola Superior de Agricultura “Luiz de Queiroz” (ESALQ), Piracicaba, SP, Brazil; 2 Department of Agronomy, Universidade Estadual do Centro-Oeste (UNICENTRO), Guarapuava, PR, Brazil; 3 Department of Biology, University of Western Ontario, London, Ontario, Canada; University of Tours, France

## Abstract

Prevailing abiotic conditions may positively or negatively impact insects at both the individual and population levels. For example while moderate rainfall and wind velocity may provide conditions that favour development, as well as movement within and between habitats, high winds and heavy rains can significantly decrease life expectancy. There is some evidence that insects adjust their behaviours associated with flight, mating and foraging in response to changes in barometric pressure. We studied changes in different mating behaviours of three taxonomically unrelated insects, the curcurbit beetle, *Diabrotica speciosa* (Coleoptera), the true armyworm moth, *Pseudaletia unipuncta* (Lepidoptera) and the potato aphid, *Macrosiphum euphorbiae* (Hemiptera), when subjected to natural or experimentally manipulated changes in atmospheric pressure. In response to decreasing barometric pressure, male beetles exhibited decreased locomotory activity in a Y-tube olfactometer with female pheromone extracts. However, when placed in close proximity to females, they exhibited reduced courtship sequences and the precopulatory period. Under the same situations, females of the true armyworm and the potato aphid exhibited significantly reduced calling behaviour. Neither the movement of male beetles nor the calling of armyworm females differed between stable and increasing atmospheric pressure conditions. However, in the case of the armyworm there was a significant decrease in the incidence of mating under rising atmospheric conditions, suggesting an effect on male behaviour. When atmospheric pressure rose, very few *M. euphorbiae* oviparae called. This was similar to the situation observed under decreasing conditions, and consequently very little mating was observed in this species except under stable conditions. All species exhibited behavioural modifications, but there were interspecific differences related to size-related flight ability and the diel periodicity of mating activity. We postulate that the observed behavioral modifications, especially under decreasing barometric pressure would reduce the probability of injury or death under adverse weather conditions.

## Introduction

Abiotic factors, such as temperature, photoperiod, wind speed and rainfall, play important roles in determining the geographic distribution and population dynamics of insect species, as well as the diel periodicity of individuals. For example, rainfall is important, with the peak populations of many insect species observed during wet-seasons [1]–[2], as precipitation directly provides water essential for development and reproduction, [3]–[5], and indirectly through increased food availability [6]. Furthermore, insects have layers of hairs and wax-coated cuticle that confer hydrophobicity [7] and a strong exoskeleton and low mass that reduces the impact of raindrops, so flight is possible in light rain [8]. Similarly, wind currents play an important role in both short and long distance movement within and between habitats [9], as well as the emission of, and responsiveness to, sex pheromones [10]. However, due to the general small size and fragile nature of insects the heavy rains and strong winds associated with storms are potentially important mortality factors [11]–[12]. Thus, adaptations allowing individuals to detect imminent changes in weather conditions would be beneficial and a limited number of studies have shown that insects [13], like mammals [14], birds [15], reptiles [16] and fish [17], modify different behaviours [18]–[22] in response to the rapid drop in atmospheric pressure (>4 mbars) in the hours preceding a storm [23].

Given the scarcity of the literature and relevance of the topic both under field conditions and when running behavioural assays in the laboratory, we examined the effect of atmospheric conditions on the courtship and mating behaviors of the curcurbit beetle, *Diabrotica speciosa* (Coleoptera: Chrysomelidae), the true armyworm moth, *Pseudaletia unipuncta* (Lepidoptera: Noctuidae), and the potato aphid *Macrosiphum euphorbiae* (Hemiptera: Aphididae). We selected species in three different Orders that vary significantly in mass, morphology, and seasonal biology, in order to test the hypothesis that changes in courtship and mating behaviors in response to changes in barometric pressure is a common phenomenon in insects.

## Materials and Methods

### Diabrotica Speciosa

We examined the response of males to an extract of the female sex pheromone using a glass Y-tube olfactometer (arms of 20 cm long and 3 cm of tube diameter) at airflow of 1 l/min for each arm, in the Laboratory of Chemical Ecology and Insect Behavior, University of São Paulo, Piracicaba, Brazil. The sex pheromone was obtained from a headspace collection of 30 virgin females and the resulting hexane extract was concentrated to 15 female equivalent [24]. Glass chambers containing filter paper incorporated with sex pheromone (150 µL or 15 female equivalent) or solvent (hexane) were connected to the ends of the olfactometer arms and position of the treatments exchanged after each replicate to avoid any positional bias. Three to six-day-old virgin males were tested in the first half of the scotophase, the period of normal mating activity [24], using a light with a red diode bulb. We determined *(i)* the proportion that exhibited general activity within 5 min (the maximum time of each assay) and *(ii)* the proportion of the active beetles that chose the arm with the female pheromone source. The assays were conducted over a series of days under constant temperature (25±1°C), humidity (60±10%) and photoperiod (12L:12D) conditions, and the hourly barometric pressure conditions obtained from the local National Institute of Meteorology (INMET station). Based on the results of a previous study on the parasitic wasp *Aphidius nigripes* [20], we examined the effects of barometric changes that occurred in the 12 h prior to the onset of the bioassay. We collected the responses when barometric conditions were considered *(i)* stable (pressure did not oscillate more than ±1.5 mbar in the 12 h prior to assays; *N = *50), *(ii)* increasing (pressure was stable for the first 6 h, but increased by at least 2 mbar within the next 6 h; *N = *30) or *(iii)* decreasing (pressure was stable for the first 6 h, but decreased by at least 2 mbar within the next 6 h; *N = *70).

In a previous study on the mating of *D. speciosa*, Nardi et al. [24] described in detail the precopulatory behavior, and reported that about 30% of males did not exhibit the full sequence of precopulatory behaviours. We wished to determine if this “reduced” behaviour was related to atmospheric pressure conditions so we compared the precopulatory behaviour and time to the onset of mating [24] of individual 3–6 day old virgin pairs, held in individual mating arenas (10×10 cm), under both the stable (*N* = 18) and falling (*N* = 26) atmospheric conditions described above.

### Pseudaletia Unipuncta

Behavioural assays on the armyworm moth were conducted inside a controlled barometric pressure chamber at the Advanced Facility for Avian Research (AFAR), at the University of Western Ontario, London, Canada. All abiotic conditions were kept constant (see below) except pressure, that was manipulated according to the categories: *(i) stable* – insects were put in the chamber at 972±1 mbar of pressure during 12 h prior to the assay; *(ii) increasing* – insects were at 972±1 mbar for 6 h and then pressure was gradually (and approximately linearly) increased to 977±1 mbar over 6 next hours; *(iii) decreasing* – insects were held at 972±1 mbar for 6 h and then the pressure decreased to 967±1 mbar over the next 6 h. The 12 h experimental manipulation of pressure was carried out just prior to the peak mating period, which for the nocturnal armyworm was in the latter half of the photophase and the first half of the scotophase.

The *P. unipuncta* assays were carried out at 25±1°, 65±10% RH under a 16L:8D photoperiodic regime, 6–8 day old virgin females were used, as sexual maturity in this species occurs a number of days post-emergence [25]. To determine the effect of atmospheric pressure on calling behaviour, females were observed in individual plastic cages (15 cm high and 5 cm diam) and fed on 8% sugar solution. Females were subjected to one of the three 12 h experimental conditions (*N = *32, 74 and 48 under decreasing, stable and rising atmospheric conditions), and the calling behaviour monitored using a flashlight with a red Wratten filter over the following 2 h, set up to coincide with the peak calling period. Females were considered to be calling (releasing the sex pheromone) when the pheromone gland was clearly exposed [25].

The same protocol was used to determine the effect of atmospheric conditions on *P. unipuncta* mating. In this case 42, 71 and 50 individual pairs of 6–8 day old virgin females and males in 20×15×15 cm cages were tested under decreasing, stable and rising atmospheric conditions, respectively. At the end of each assay females were removed, dissected and mating confirmed by the presence of a spermatophore.

### Macrosiphum Euphorbiae

The experiments examining the effect of atmospheric pressure on calling behaviour of *M. euphorbiae* oviparae were carried out at 20±1°C under a 12L:12D photoperiodic regime, as this species reproduces parthenogenetically throughout the summer and sexual reproduction only occurs in the fall. As the aphid is diurnal, the 12 h period over which oviparae were subjected to the changes in atmospheric pressure occurred during most of the scotophase and the first hours of the photophase. Three-day-old virgin oviparae (*N = *36, 21, 30 under decreasing, stable and rising atmospheric conditions) were placed in individual plastic cages (15 cm high, 5 cm diam) with a freshly cut potato leaf in water. Pairs were observed during the early photophase, when the peak occurs [26]. A female was considered as calling when it raised her abdomen and the third pair of legs off the substrate [26].

The effect of atmospheric pressure on mating in aphids was determined using a similar protocol as the one used for the calling behaviour of oviparae, but, in this case testing individual virgin pairs of 1-day-old males and 3-day-old oviparae. There were 36, 21 and 30 pairs under decreasing, stable and rising atmospheric conditions, respectively.

### Statistical Analysis

Comparisons of the proportions of *D. speciosa* males responding to a female pheromone source, the proportions of *P. unipuncta* and *M. euphorbiae* females that exibited calling behaviour, as well as the incidence of mating in all three species under the different atmospheric conditions were carried out using chi-square analyses. The time from first contact of *D. speciosa* males with a receptive female until the insertion of aedeagus under stable and decreasing pressure was compared using Mann-Whitney U test. Statistical analyses were conducted in *R* package software 2.1.8.

## Results

**Figure 1:**
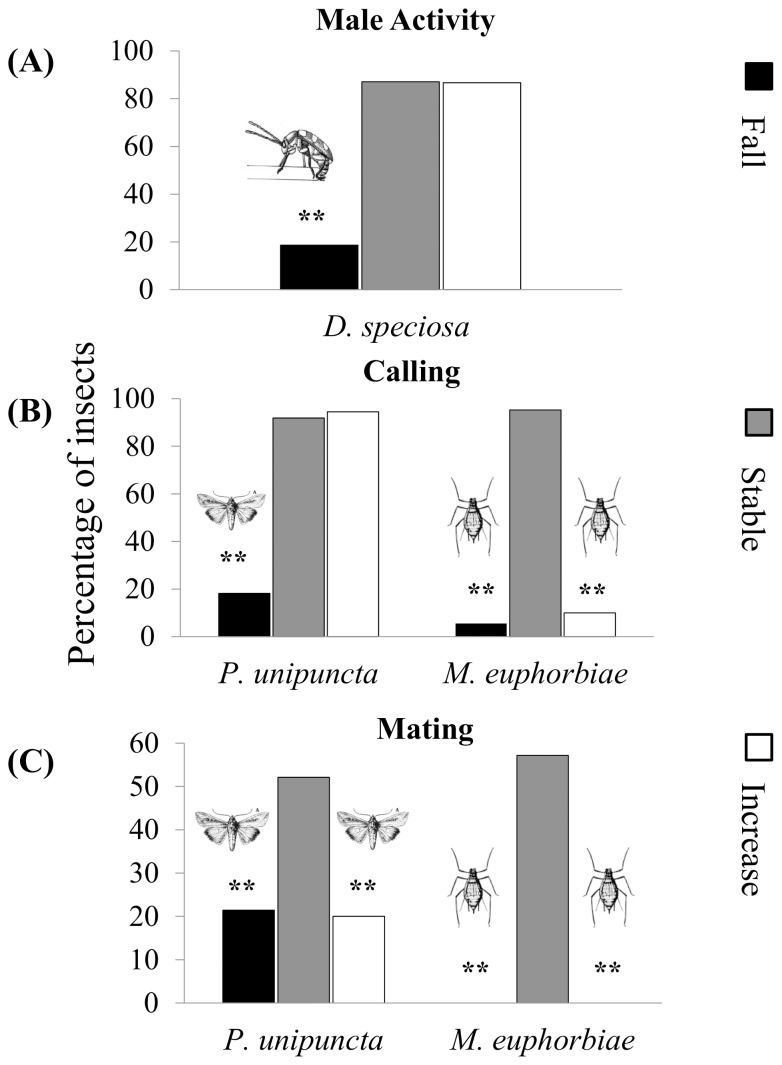
Insect Behaviour under different barometric pressure conditions. Frequency of different behaviours exhibited by three different insect species when subjected to stable, decreasing and increasing barometric pressure. A: The proportion of active *D. speciosa* males in Y-tube olfactometer to sex pheromone stimuli; B: The proportion of *P. unipuncta* and *M. euphorbiae* females exhibiting calling behaviour; C: The proportion of *P. unipuncta* and *M. euphorbiae* couples mating. **Indicates significant difference among treatments at 1%.

The locomotory activity of *D. speciosa* males in the Y-tube olfactometer with female pheromone extracts was affected by barometric pressure (Figure 1A, df = 2, *χ^2^ = *13.38, *P*<0.001), being significantly lower when pressure decreased compared to stable or increasing pressure conditions (Figure 1A, stable *vs.* decreasing df = 1, *χ^2^ = *56.38, *P*<0.001; increasing *vs.* decreasing df = 1, *χ^2^ = *40.93, *P*<0.001). The proportion of active beetles that selected the arm containing the female sex pheromone were 68, 62 and 85% under falling, stable and rising pressure conditions, respectively. There was an overall effect of atmospheric pressure on the male response for the pheromone (df = 2; *χ^2^* = 14.02, *P = *0.001), being more intense under conditions of rising pressure (df = 1; *χ^2^* = 6.23, *P*<0.01).

The time between the male first contacting the female and intromission was significantly shorter when atmospheric pressure was falling than when it was stable (Figure S1, 5.2±1.2 min *vs.* 16.2±3.1 min; *U* = 364, *P* = 0.001). This is, in large part, due to the fact that under declining pressure conditions 16/26 (63%) of the males immediately mounted the female without any intermediate courtship behaviours, while all of all males under stable conditions (18/18) exhibited pre-copulatory behaviours such as antennation (Figure S1).

Armyworm female calling behaviour was also altered by variation of barometric pressure (Figure 1B, df = 2, *χ^2^* = 82.47, *P<*0.001), with fewer females calling when the pressure dropped than under stable or increasing pressure conditions (Figure 1B, stable *vs.* decreasing, df = 1, *χ^2^* = 56.71, *P*<0.01; increasing *vs.* decreasing df = 1, *χ^2^* = 50.15, *P*<0.01). Similarly, the incidence of mating observed was affected by barometric pressure (Figure 1C, df = 2, *χ^2^* = 17.60, *P<*0.001). However, in contrast to female calling that was only affected by a decrease in pressure, the incidence of mating was significantly lower when barometric pressure fell or rose (Figure 1C, stable *vs.* decreasing df = 1, *χ^2^* = 10.29, *P<*0.001; stable *vs.* increasing df = 1, *χ^2^* = 12.74, *P*<0.01).

The proportion of potato aphid oviparae calling was significantly altered in response to barometric pressure changes (Figure 1B; df = 2, *χ^2^* = 59.94, *P<*0.001), being significantly lower when air pressure rose or fell than under stable pressure conditions (Figure 1B, stable *vs.* decreasing df = 1, χ^2^ = 45.01, *P<*0.001; stable *vs.* increasing df = 1, *χ^2^* = 36.25, *P*<0.01). A similar pattern was also observed with respect to mating success (Fig. 1C; df = 2, χ^2^ = 43.75, *P<*0.001; stable *vs.* decreasing df = 1, *χ^2^* = 22.42, *P<*0.001; stable *vs.* increasing df = 1, *χ^2^* = 36.25, *P*<0.01).

## Discussion

The atmospheric conditions tested in our experiments are well within the range changes in atmospheric pressure that insects would encounter under natural field conditions. A rise in atmospheric pressure would generally be associated with clear weather and moderately strong winds, while a drop in atmospheric pressure would be associated with inclement weather, including high winds and possible rainstorms which for insects can result in high mortality [27]. Our results clearly show that changes in atmospheric pressure in the 12 h preceding the assays significantly affect behaviours associated with mating in all three species tested, although noticeable interspecific differences were observed.

The majority of research examining climatic conditions on *Diabrotica* spp. has been carried out on the Western Corn Rootworm, *D. virgifera virgifera*. There is strong evidence that both males and females are capable of flying considerable distances throughout their habitat in search of resources such as food and mates [28]–[30]. Furthermore there are two distinct periods of activity in the morning and late afternoon, which coincide with the diel emergence patterns of both sexes [31]. While biotic factors, such as egg load in females [32] influence flight activity, prevailing climatic conditions also play a major role [30], [31], [33]–[35] in both the intensity and timing of flight. Specifically, flight activity is markedly reduced at low (<15°C) and high (>32°C) temperatures, at wind velocities >2 m/s and when it is raining, such that atmospheric conditions substantially reduce flight during 30% of the days along the study [33]. In a study examining curcurbitacin baits for Diabroticina in the United States and Argentina (in the latter site *D*. *speciosa* represented >90% of all beetles caught) an analysis of weather data showed that periods of low trap catch were associated with low atmospheric pressure conditions [34].

Thus, the significant decrease in general activity observed with *D*. *speciosa* males in the olfactometer assays under decreasing atmospheric pressure would certainly help explain the patterns of flight activity observed in previous studies, as lowered activity should reduce the probability of injury or death from exposure to strong winds and rain. While there may be moderate to strong winds occurring under rising pressure conditions when the weather is good, these are more frequent during periods of atmospheric instability occurring from mid-morning to late afternoon because of solar radiation. It has been postulated that these conditions actually facilitate long distance dispersal of beetles [28] and would explain why there was no difference in *D*. *speciosa* male activity between stable and rising conditions.

Given the results of the Y-tube assays it would initially seem somewhat surprising that *D*. *speciosa* males actually mated when placed in close proximity (<10 cm) of virgin females under decreasing pressure conditions. However, this modified behaviour could be a response to a perceived reduction in life expectancy, as reported for parasitic wasp, *Leptopilina heterotoma* [36]. This idea is supported by the fact that more than 60% of males did not exhibit precopulatory behaviours while all males tested under stable conditions did. This possibility certainly needs to be investigated in more detail, including looking at possible changes in female behaviour, as they did not reject males showing no courtship behaviours.

A similar pattern of response was seen with the calling behaviour of virgin female armyworm moths, with a significant reduction in calling only observed under decreasing atmospheric conditions. Females do not fly while calling but select positions on the host plants that facilitate the diffusion of the pheromone plume [37] and being in these more exposed sites would increase the risk of injury or mortality under rainy and windy conditions. Understandably, as fewer females were calling there was also a decrease in the incidence of mating under the decreasing atmospheric conditions. However, while female calling was similar under stable and rising atmospheric conditions, the incidence of mating was significantly lower under conditions of increasing air pressure suggesting a change in male responsiveness. Males fly while searching for receptive females but while some wind is needed to move the pheromone plume, male responsiveness to pheromone decreases with increasing wind speed [38]. Thus, while wind turbulence is lower at night in the absence of solar radiation males may associate certain conditions of rising atmospheric pressure with those that could reduce life expectancy, and consequently modify their behaviour.

Unlike the nocturnal virgin females of *P. unipuncta,* the diurnal oviparae of *M. euphorbiae* showed a marked reduction in calling behaviour under both increasing and decreasing atmospheric pressure conditions. However, this is not particularly surprising when one considers that sexually mature potato aphid oviparae move to the edge of the leaf and raise their metathoracic legs off the plant, as the pheromone is released from the hind tibia [26]. In such a position, the small, wingless oviparae only have four of their six legs grasping the plant and thus would be exposed to turbulent winds whenever there were changing atmospheric conditions. Consequently, there would have a high probability of being dislodged, resulting in a low probability of relocating a suitable overwintering host plant, and an increased incidence of mortality. There was no mating under conditions of either increasing or decreasing atmospheric conditions, and obviously this was due in part to the low number of calling females. However, it is not unreasonable to think that males also respond to the changes in atmospheric pressure for the same reasons suggested for females. Winged male are very poor fliers and flight initiation is inhibited at low windspeeds [39], so once blown off the plant they would have little chance to relocate overwintering hosts with receptive oviparae.

The results presented show that three very different insect species all modify aspects of their sexual behaviour in response to changing barometric pressure. However, there is a great deal of interspecifc variability in their responses that can be related to differences in size, flight ability and the diel periodicity of mating. These data, together with the few examples in the literature, suggest that changes in foraging and mating behaviours in response to changing barometric pressure are common in insects. However, in order to develop general theories that would be useful in understanding both individual behaviours and the resulting population trends, a greater diversity of species with markedly different ecologies need to be investigated. It would also be of considerable interest to examine interspecific variability for species with a wide geographic range as they may vary as a function of prevailing ecological conditions, as seen in the defence responses of the pea aphid [40]. Another fruitful area of research would be to determine exactly how changes in atmospheric pressure are detected and integrated. It has been suggested that external mechanoreceptors are involved [41], specifically cuticular filiform sensilla which are sensitive to faint air movements, and changes in the force of air weight result in deflection of sensilla, directly transmitting the stimulus to dendrite tip [42]. A more recent study has also implicated hygroreceptors, which can detect forces imposed on the dendritic membrane due to air pressure changes [43]. Other potential means of assessment also need to be investigated, for it has been suggested that changes in the size of air bubbles present in the foregut allow bark beetles to detect fluctuations in atmospheric pressure and consequently modulate their response to aggregation pheromones [44].

## Supporting Information

Figure S1 **Ethogram of** ***Diabrotica speciosa*** **courtship under different barometric pressure conditions.** Percentages of *D. speciosa* displaying the different steps of courtship, under stable (full line) and decreasing (dotted line) barometric pressure. *indicates significant difference.
(TIF)
