# Supplementary figures and images for: Weather Forecasting by Insects: Modified Sexual Behaviour in Response to Atmospheric Pressure Changes
Source: PLoS One. 2013 Oct 2;8(10):e75004. doi: 10.1371/journal.pone.0075004 (PMC3788776; doi:10.1371/journal.pone.0075004)

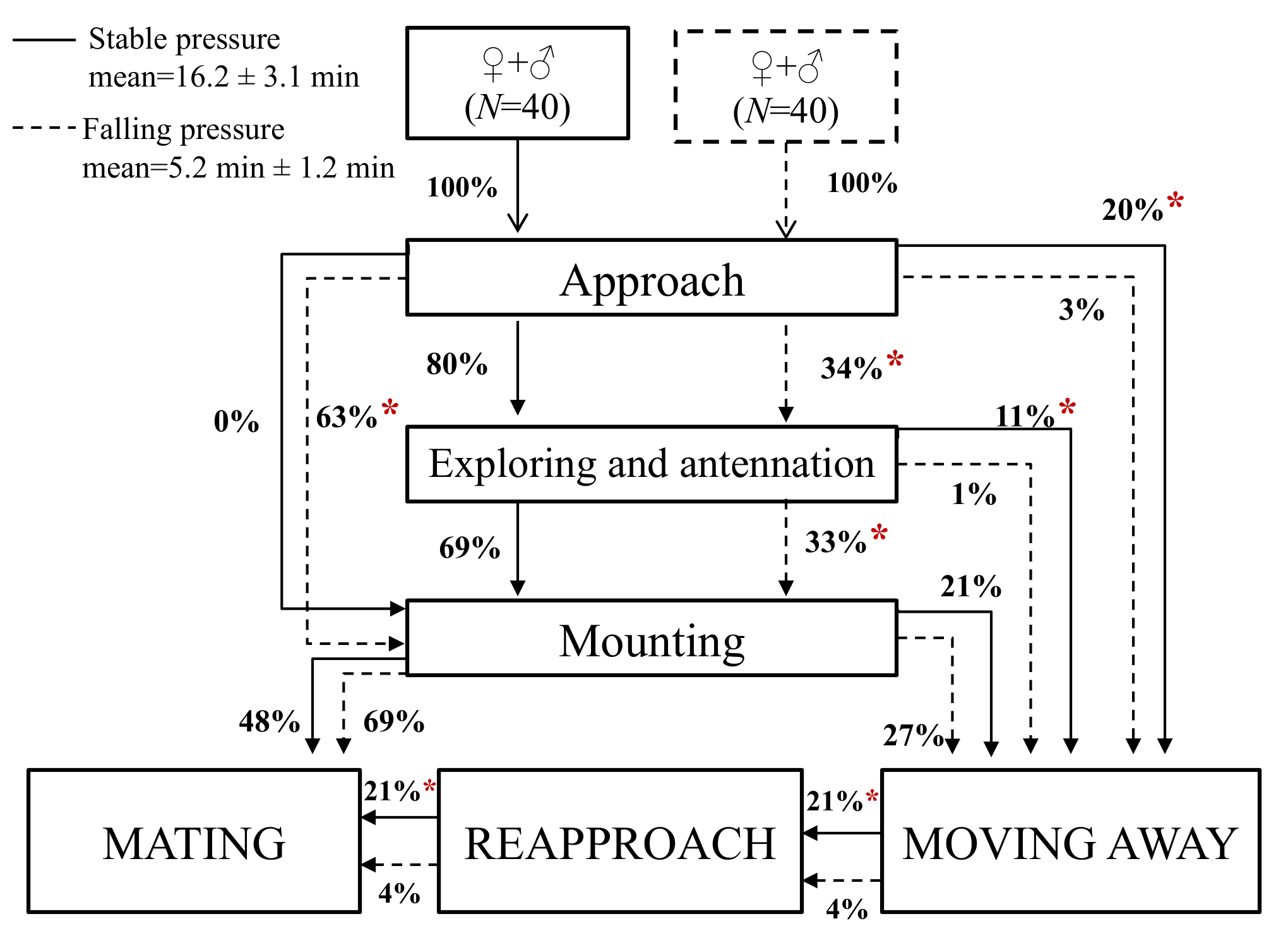

Supplement: Figure S1 — Ethogram of Diabrotica speciosa courtship under different barometric pressure conditions. Percentages of D. speciosa displaying the different steps of courtship, under stable (full line) and decreasing (dotted line) barometric pressure. *indicates significant difference. (TIF) [file pone.0075004.s001.tif]
